# Monthly Summary

**Published:** 1893-11

**Authors:** 


### Monthly Summary.

NERVOUS EXHAUSTION.—About twenty-five years ago nervous exhaustion was called by Dr. Beard an "American disease," because the affection was much more prevalent in the United States than elsewhere.

It has been stated—and no doubt correctly—that it is not only a product of civilization, but of civilization run mad—crazed by its everlasting rush.

It is a well-known fact that the body is in a perpetual state of aasimilation and elimination—nutrition and waste—and that the two processes balance each other in a healthy and normal physical condition; that unless the waste product is regularly carried off, the system is poisoned by its accumulation. It is this self-poisoning which, for instance, brings on the fatal results of Bright's disease, etc.

The cells are the ultimate nutrients of the body, whether they are of the muscles, membranes, nerves or cerebral substance. They select the appropriate nourishment from the blood, assimilate it, and cast off the debris or waste material, which is always fatal if retained in too large quantities.

The nervous system is constantly in action—it is never fully at rest; and the cells of every portion of the body must be kept incessantly at work. While this work is going on there is a formation of waste product which, in normal action, is taken care of and duly eliminated; but in prolonged excessive mental activity the waste accumulates and acts as a poison to the nerves themselves, interfering with their normal action.

Slight disturbances of the nervous functions can be easily remedied, but excessive irritability and weakness of the nervous system demand our most careful consideration and attention.—*The Medical Summary.*

---

THE BRIDGE-WORK FAD.—By Dr. S. F. Gilmore, Princeton, Ind.—This is an age of "fads," even in dentistry.

Perhape the patrons of bridge-workmen are the true sufferers, judging from cases that have come under my observations, both in this country and in Europe in the past few years. In looking for a young man to take care of my office, I found that it was a current belief among those with whom I corresponded or came in contact, that the high standard of their ability should be inferred from the fact that they could make bridge-work. I would consider it much more satisfactory evidence of a man's attainments if he were to assure me that he could "stop tooth-ache." For this is therapeutical, and the "bedrock of the profession. I do not wish to convey the idea that I am opposed to inserting bridges, for I have made a great many that are doing good service, but let it be understood that this class of work is merely adjunctive, and one of the simplest operations in dentistry.

Let a man seek with heart and mind the knowledge necessary to enable him to diagnose, following the accomplishment of this art, with the ability to apply or prescribe the remedies indicated.

I would classify attainments in the following order:

1st. Therapeutics.

2nd. The selection and insertion of the proper materials for arresting decay in carious teeth.

3rd. The selection and artistic arrangement of a set of artificial teeth.

4th. If he is able to judge of favorable conditions for a bridge—make it.

While in the office of a neighboring dentist a few days ago, a lady called to consult him, or rather to have him relieve her of a $200 bridge that had been mounted a few weeks before, on six or seven diseased teeth and roots, by another dentist, who styles himself a bridge-workman. The mechanical part of the work was good. but a maker of artificial limbs might, with as much propriety, cement a wooden leg on a gangrenous stump.—*Dominion Dental Journal.*

---

CHAS. ELROY ON HOW TO TREAT COCAINE POISONING. Your first duty is to prevent syncope, afterward to combat respiratory and cardiac collapse. The therapeutic means of doing this are unfortunately very few. At the very beginning place the patient in a perfectly horizontal position, which will diminish the force of the syncopal condition. Sprinkle ice-water over the face, and to prevent convulsions envelop the body in cloths wrung out of cold water.

If asphyxia threatens, practice flagellations with wet towes, massage, artificial respiration.

Against tetanization of the respiratory muscles give inhalations of chloroform.

Where there is great pallor, provoke vaso-dilatation, modify the arterial pressure, and diminish the encumberment of the central circulation, by the administration of amyl nitrate (by inhalation.)

If these means prove ineffectual, and deglutition is impossible, give hypodermic injections of caffein, and of sulphuric ether (15, 30, even 45 minims).

In a word, bend your efforts toward moderation of reflex excitability of the nervous system, sustain the heart, and re-establish the equilibrium of the blood-pressure. The treatment of acute cocaine-intoxication is particularly and above all a case for arterial medication.

Commenting on the foregoing, M. Choupe ("Bulletin Medical) counsels in addition the use of hypodermic injections of morphin. These should be given only in the very outset, however, and should be only sufficiently large to produce the physiological effects of the drug, say from one-half to five-eighths of a grain.—*Revue de Clinique.*

---

FOREIGN DIPLOMAS IN GREAT BRITAIN.—It is a common observation that nowhere in the world have foreigners more liberty, both civil and religious, than in old England. Occasionally some malicious boor who construes this word into the privilege of spitting, smoking, and generally misbehaving wherever he may desire, sends forth to the world his impressions of the old sod as the most down-trodden land on the face of the earth; but no people have been more just and generous in their judgments, more fair or frank in their estimate of the British character, than American writers and travellers.

Some—not all—of our contemporaries over the border, however, seem unable dispassionately to discuss the action of the General Medical Council in revoking the privilege heretofore awarded certain foreign dental colleges, while refusing any similar favor to foreign medical schools. The worthy editor of the *Review* makes it a personally national matter, and heads one of his recent editorials, "No American need apply."

It was always a mystery to us why the Council was so invidious as to discriminate in favor of two of the American schools against others which were equally reputable, especially when the Council was well aware when the choice was made that not even the two favored came up to the required standard. It looked like favoritism, yet we do not see, in spite of the inconsistencies exhibited, that the action of the Council was specially against American colleges. It happened to be brought about by reason of the preferrence given in the past to two American schools; but it is possible to reason the matter coolly, and accept it in the spirit of justice to the British student and practitioner, who certainly have the first claim to consideration, otherwise it imposes a penalty upon the Briton, and holds forth a premium to foreign education.

It may surprise our American cousins to know that we Canadian dentists have no recognition whatever in the old home, and this in spite of the fact that in two of the provinces our matriculation is fully up to the standard required; and that for over twenty years we have had precisely the same system of indentured apprenticeship of four years of twelve months each year, with compulsory attendance upon Anatomy (theoretical and practical), Physiology and Chemistry, and primary and final examinations before boards of examiners elected by the profession. Brother Jonathan was getting favors refused to John Bull's own kids.

One instance may be here mentioned as affecting Canadians, Australians, and other loyal subjects of the Empire. One of our Canadian students, born within the sound of Bow-bells, and desiring to return to England to practice, passed the very highest matriculation recognized in England, and graduated after three full courses at Harvard. He selected Harvard because he believed his diploma would be recognized. He then proceeded to London, presented the necessary papers to the Council, but was told that the law gave him no "privilege," *as he was a British subject.* In fact, as a further instance of the unfairness of the Act to British subjects, he was positively refused a privilege granted a moment after to a friend from New York. He could get over the difficulty by forswearing his allegiance to his sovereign and becoming a citizen of a foreign nation !—*Dominion Dental Joarnal.*

---

F. PETERSON ON CLONIC SPASM OF THE MUSCLES OF MASTICATION.—Patient a woman, aged fifty-seven, who six years before had had all her upper teeth removed and artificial ones put in. The first set did not fit well, and a new one was substituted. The work about the mouth, and the necessity for keeping her mouth open for long periods of time while she was in the dentist's chair, resulted in the development of this spasm. When she was sitting quietly, not using the jaw muscles, there was a continuous clonic spasm of the masseters, temporals and pterygoids. The jaw opened and shut slightly and moved from side to side. She was tired and worn out with trying to keep her teeth together. The chief difficulty, however, was when she attempted to speak; then the mouth opened wide and there was a subluxation of the jaw downward and forward from the glenoid cavity. During the first six months the mouth would not close at all, except at night, when the spasm relaxed.

Dr. Peterson says that while clonic spasm of the masticatory muscles or trismus was quite a common symptom, the condition presented in this case was very rare. As regarded treatment, atropine, hyoscine, conium, and electricity had been used perseveringly without any special effect. Latterly sulphate of duboisine, in doses of one two-hundredth of a grain, three times daily, had afforded much relief by quieting the spasmodic movements almost wholly at times. In addition she wore an apparatus made especially for her, which kept her jaw closed and allowed her to talk between her teeth without the uncomfortable clonic spasm of the depressors of the jaw, although the chronic movements of the masseters and pterygoids might keep on as before. The movements ceased at night. The affection had lasted nearly seven years.—*Southern Dental Journal.*

---

PHAGOCYTOSIS.—Dr. A. Looss replies to the reply of Prof. Metschnikoff on the great subject of how the tadpole's tail disappears. According to Metschnikoff it is due to phagocytosis, and phagocytes which digest the contractile substance are formed from the sarcoplasm and muscle nuclei.

It was this definition of the phagocyte which started the controversy; for, according to the author, as generally accepted, and that too from Metschnikoff himself, who formulated the doctrine, the phagocyte was an amœboid connective-tissue cell or a mobile lymph or blood-corpuscle. It was therefore surprising to discover that the phagocytes had quite a different origin, and to find it laid down that their own inventor had never identified them with leucocytes. It was suggested to the author that his preparations were unsatisfactory and his interpretation of the facts erroneous.

It was only natural to reply, and the author places in parallel columns extracts from the earlier and later writings of Prof. Metschnikoff, showing how the views of the latter have materially altered. For example the following are contrasted :—

"At the beginning of the metamorphosis ambœboid cells accumulate in the vicinity of some tail-muscles," and yet neither in the muscle itself nor in its neighborhood are ever perceived any agglomerations of leucocytes. It is hardly possible to avoid the conclusion that a phagocyte may mean almost any kind of cell.—*Royal Microscopical Society.*

---

MR. HUMBY, speaking of pyorrhœa alveolaris, said that he thought that the cause was generally mistaken; he thought that the disease was caused by death of a portion of the cementum in the root, and the lacunæ became charged with septic material, and thus set up irritation of the gum and absorption of the alveolus, and thought that if some drug was used that would coagulate the albuminous contents of the lacunæ, the disease might be arrested; and he mentioned a case in practice in which he had successfully used nitrate of silver, thirty grains to the ounce.—*British Journal.*

---

IT IS the opinion of Dr. Rhein that "where gold is added to amalgam it does not produce a disintegrating effect, but makes a gold amalgam. The addition of amalgam to gold is different, because of the affinity of the mercury in the amalgam for the gold acting continuously, destroying and disintegrating the homogenous qualities of the gold, but not producing a perfect amalgamation."—*Southern Dental Jour.*

EUCALYPTOL, the ethereal oil of eucalyptus, has a peculiar action on the suppurative process. It paralyzes, as has been ascertained, the white corpuscles as soon as they have penetrated the blood vessel wall during inflammation. The process of tissue disintegration is hence checked by this drug. —*Southern Dental Journal.*

---

W. K. VANDERBILT was not feeling quite well the other day, so he decided to take a trip to Europe, accompanied by his physician. The doctor said he could not afford to leave his practice, which was worth $1,000 a week. Mr. Vanderbilt offered to give him $10,000 to make the trip of six weeks with him. They went.

---

MANY a child in after years would be saved from untold suffering if parents had paid more attention to teeth during early childhood. Rather let the face or hands of your children remain unwashed than to allow them to suffer from unclean teeth. *Let every parent remember this.*

---

THE LAST STRAW.—Judge—This dentist says you requested him to pull your tooth, and after he had done so, got out of the chair and knocked him down.

Treetop—That's right; but I didn't "request" him to ask me if "it hurt," consarn him!—*Truth.*

